# Elevation of NR4A3 Expression and Its Possible Role in Modulating Insulin Expression in the Pancreatic Beta Cell

**DOI:** 10.1371/journal.pone.0091462

**Published:** 2014-03-17

**Authors:** Weina Gao, Yuchang Fu, Cong Yu, Shunke Wang, Yuchao Zhang, Chen Zong, Tongfu Xu, Yong Liu, Xia Li, Xiangdong Wang

**Affiliations:** 1 Department of Cell Biology, Shandong University School of Medicine, Jinan, China; 2 School of Medical Technology and Engineering, Henan University of Science and Technology, Luoyang, China; 3 Department of Nutrition Sciences, University of Alabama at Birmingham, Birmingham, Alabama, United States of America; 4 The Institute for Nutritional Sciences, Chinese Academy of Science, Shanghai, China; 5 Key Laboratory of Protein Sciences for Chronic Degenerative Diseases in Universities of Shandong (Shandong University), Jinan, China; Baylor College of Medicine, United States of America

## Abstract

**Background:**

NR4A3/NOR-1 is a member of the NR4A orphan nuclear receptor subfamily, which contains early response genes that sense and respond to a variety of stimuli in the cellular environment. The role of NR4A3 in insulin expression in pancreatic beta cells remains unknown.

**Methods:**

Dynamic changes in NR4A3 were examined in a pancreatic beta-cell line, MIN6, treated with thapsigargin (TG), palmitate (PA), tunicamycin (TM), and dithiothreitol (DTT), chemicals that produce cell stress and even apoptosis. We exploited virus infection techniques to induce expression of NR4A3 or three deletion mutants, and determined expression of insulin and insulin regulatory genes in MIN6 cells.

**Results:**

TG and PA, two endoplasmic reticulum (ER) stress inducers, were able to induce unfolded protein response (UPR) activation and elevation of NR4A3 expression in MIN6 cells, whereas TM and DTT, two other ER stress inducers, were able to induce UPR activation but not NR4A3 elevation. MIN6 cells over-expressing NR4A3 protein after adenoviral infection exhibited reduced transcription of the insulin genes *Ins1* and *Ins2*, and reduced insulin protein secretion, which were negatively correlated with NR4A3 expression levels. Functional analysis of different deletion mutants of NR4A3 showed that deleting the activation domain AF1 or the DNA-binding domain abolished the down-regulation of insulin transcription by NR4A3 in MIN6 cells, indicating that this down-regulative role was closely related to the NR4A3 trans-activation activity. Over-expression of NR4A3 in MIN6 cells resulted in reduced mRNA transcription of the insulin positive-regulation genes, *Pdx1* and *NeuroD1*.

**Conclusion:**

Some ER stress inducers, such as TG or PA, are able to elevate NR4A3 expression in MIN6 cells, while others, such as TM or DTT, are not. Over-expression of NR4A3 in MIN6 cells results in down-regulation of insulin gene transcription and insulin secretion. NR4A3 reduces insulin gene expression by modulating the expression of *Pdx1* and *NeuroD1*.

## Introduction

Nuclear receptors make up a large family of ligand-dependent transcription factors [1]. They have common structural motifs: a highly variable NH_3_-terminal region that contains a ligand- independent activation domain called AF1; the central DNA-binding domain (DBD), consisting of two highly conserved zinc-finger motifs, which targets the receptor to specific DNA sequences called hormone response elements (HRE); and the C-terminal ligand-binding domain (LBD), which functions in ligand recognition, receptor dimerization, and cofactor interaction [2]. Based on the DBD sequence homology to confirmed nuclear receptor, such as glucocorticoid receptor (GR), a number of orphan nuclear receptors were found, whose natural or synthetic ligand were not been identified initially [3]. Some adopted orphan receptors' ligands have been identified in subsequent researches, for example the ligand of Peroxisome proliferator-activated receptors (PPARs) is fatty acids (FA) [4]. The NR4A subfamily is true orphan receptor because of its ligand binding pocket (LBP) is entirely filled by hydrophobic amino acid side chains, and lacks a cavity for ligand binding [5].

NR4A1 (also known as Nur77, TR3, or NGFI-B), NR4A2 (also known as Nurr1, RNR-1, or TONOR), and NR4A3 (also known as NOR-1 or MINOR) comprise the NR4A subfamily of orphan nuclear receptors [6]. NR4A1 was found first and its cDNA was cloned. It was regarded as one of the rapid but transient response genes induced by nerve growth factor (NGF) in the rat pheochromocytoma cell line, PC12 [7], [8]. The cDNAs of NR4A2 and NR4A3 were then cloned in succession [9], [10]. The three members of the NR4A subfamily have high levels of homology in the DBD domain (∼91 to 95%) and modest levels of homology in the LBD domain (∼60%), whereas they are very divergent in the activation domain [11], [12]. All three members were found in the nucleus, as their DBDs also contain a nuclear localization signal (NLS) [13].

Nuclear receptors such as glucocorticoid receptor (GR) act as transcriptional switches responding to ligand binding [4], but the NR4A subfamily acts mainly through increased transcriptional expression induced by exogenous stimulation. NR4A subfamily members are immediate early response genes that sense and respond to changes in the cellular environment. Many stimuli such as NGF, platelet growth factor (PGF), epidermal growth factor (EGF), serum [7]–[10], tetradecanoylphorbol-1,3-acetate (TPA), thapsigargin, dietary long-chain free fatty acids (FFA), low density lipoprotein (LDL), and 6-[3-(1-adamantyl)-4-hydroxyphenyl] -2-naphthalene carboxylic acid (AHPN or CD437) [14]–[18], have been shown to induce NR4A mRNA transcription in many cells.

NR4A have been shown to modulate cell apoptosis. In response to apoptotic stimuli in many tumor cells, induced NR4A1 and NR4A3 were able to translocate from the nucleus to mitochondria, and to interact with and produce conformational changes in Bcl-2, resulting in the conversion of Bcl-2 from a protector to a killer molecule, which leading to release of cytochrome c and apoptosis of cells [19]–[21]. The mechanism of negative selection in T-cell was closely related to NR4A translocation-regulated apoptosis [22], [23].

Studies have shown that induced NR4A1 can translocate to the endoplasmic reticulum (ER) and interact with proteins in the ER, leading to Ca^2+^ depletion and expression of the proapoptotic transcriptional regulator CHOP, resulting in ER stress-induced apoptosis [24], [25].

The NR4A subfamily has been reported to have effects on anti-apoptosis because of the roles of its members as transcription factors. NR4A1/Nur77 over-expression was shown to lead to enhanced nuclear factor (NF)-κB activity and NF-κB-dependent induction of the anti-apoptotic gene *cIAP1* in HEK293 cells, which promoted resistance to programmed cell death induced by a number of apoptosis-inducing agents [26]. NR4A2/Nurr1 was shown to interact with p53 and suppress its transcriptional activity, resulting in down-regulation of expression of the proapoptotic protein Bax in HEK293, N2a, and HCT116 p53^+/+^ cells [27].

Recent studies showed that NR4A subfamily members also have regulatory functions in metabolic tissues (including skeletal muscle, adipose tissue, and liver cells and tissues, among others) [28]–[31]. The NR4A also function as sensors in regulating the expression of a number of downstream genes. For example, NR4A1/Nur77 was shown to act as a lipotoxicity sensor in regulating glucose-induced insulin secretion in pancreatic beta cells, and inhibited transcription of insulin genes by interacting with FoxO1 [32]. NR4A3 represent a novel candidate gene for beta-cell function because common genetic variation within the NR4A3 locus determines insulin secretion [33]. The functions of NR4A1/Nur77 and NR4A3/NOR-1 appear to be redundant [34].

In pancreatic cells, the balance between ER stress and activation of the unfolded protein response (UPR) determines the fate of these cells. We designed the current study to clarify whether some ER stress inducers are able to induce expression of NR4A3, and to investigate whether enhanced expression of NR4A3 correlates with ER stress or UPR activation. We also investigated the effect of NR4A3 expression on insulin transcription and secretion. In order to explore whether NR4A3 has an effect on insulin expression in pancreatic beta cells, viral infection was used to produce stable or transient expression of NR4A3 in the MIN6 cell line.

## Materials and Methods

### Reagents and antibodies

The cell culture medium and fetal bovine serum (FBS) were purchased from Hyclone (Thermo Fisher Scientific Inc., Bremen, Germany); blasticidin S HCl (R210-01) was from Invitrogen (Life Technologies Co., San Diego, CA, USA); all restriction endonucleases were from New England BioLabs (Beijing) LTD. ; and thapsigargin (TG) (T-9033), tunicamycin (TM) (T-7765), dithiothreitol (DTT), and sodium palmitate (PA) (P-9767) were from Sigma (St. Louis, MO, USA). Unless otherwise specified, all other chemical reagents were from Sinopharm Chemical Reagent Co., Ltd.

Anti-NR4A3 monoclonal antibody (PP-H7833-00) was purchased from R&D Systems; NOR-1 (sc-30154) rabbit polyclonal antibody was from Santa Cruz Biotechnology Inc. (Santa Cruz, CA, USA); and HA-tagged antibody (TA-04), beta-actin antibody (TA-09), and all secondary horseradish peroxidase-conjugated antibodies were from Zhongshan Goldenbridge Biotechnology Co (Beijing, China).

### Cell culture

The mouse pancreatic beta-cell line, MIN6, was purchased from ATCC and grown in Dulbecco's modified Eagle's medium (DMEM), supplemented with 10% FBS, 50 µM beta-mercaptoethanol, 100 µg/ml streptomycin, and 100 U/ml penicillin at 37°C in a humidified atmosphere composed of 95% air and 5% CO_2_.

### Insulin secretion assay

Cells were seeded in 24-well plates, and cultured for 48 h. After adenovirus infection for 44 h or treatment with 0.5 µM TG for 1 h and 0.5 mM PA for 12 h, the medium was removed, and cells were washed once with HEPES-balanced Krebs-Ringer bicarbonate buffer (HKRB: 119 mM NaCI, 4.74 mM KC1, 2.54 mM CaC1_2_, 1.19 mM MgC1_2_, 1.19 mM KH_2_PO_4_, 25 mM NaHCO_3_, and 10 mM HEPES pH 7.4) without glucose. Next, cells were pre-incubated for 1 h in HKRB with 0.5% BSA and 5 mM glucose. After washing once with HKRB, cells were incubated for 2 h in 150 µl HKRB supplemented with 0.5% BSA and 25 mM glucose. The media were then collected and assayed for immunoreactive insulin by radioimmunoassay (RIA) with [^125^] iodine using an Insulin Radioimmunoassay Kit (Beijing North Institute of Biological Technology). To each well, 200 µl of 1 M NaOH was added to solubilize the cells before determination of cellular protein content using a BCA Protein Assay Kit (Sangon Biotech Co., Ltd, Shanghai, China).

### Reverse transcription PCR and real-time quantitative PCR assay

Total RNA was isolated from cultured cells using RNAiso Plus (D9108B, TaKaRa, Japan). A ReverTra Ace qPCR RT Kit (FSQ-101, TOYOBO, Japan) was used for mRNA reverse transcription (RT) according to the manufacturer's instructions. In brief, RT was performed at 37°C for 15 min in a final volume of 20 µl containing 4 µg DNase I-treated total RNA, 4 µl 5× RT buffer, 1 µl Enzyme Mix, and 1 µl Primer Mix, made up to a volume of 20 µl with nuclease-free water, then enzyme deactivation was carried out at 98°C for 5 min.

PCR was conducted using the DreamTaq Green PCR Master Mix according to the manufacturer's instructions (K1082, Thermo Scientific, Bremen, Germany). All experiments were performed at least in triplicate. The relative semi-quantitative optical density ratio was calculated using NIH Image J software, with beta-actin as an internal control.

Real-time quantitative PCR (qPCR) was performed using a CFX96™ Real-Time PCR System (Bio-Rad) and UltraSYBR Mixture (with ROX) (CW0956, Cowin Biotech, Beijing, China). The thermal cycling conditions were: 10 min at 95°C for enzyme activation; followed by 40 cycles of denaturation for 30 s at 95°C, and 1 min of annealing and extension at 60°C. Relative gene expression was calculated by the ^ΔΔ^Ct method. Final results were expressed as the fold difference in gene expression normalized to beta-actin and relative to the control conditions. All experiments were performed at least in triplicate.

The primers used in the experiments are shown in Table S1.

### Western blotting

Cells were washed with ice-cold phosphate-buffered saline (PBS) in the presence of 0.4 mM sodium orthovanadate. RIPA lysis buffer [0.1% SDS, 1% TritonX-100, 150 mM NaCl, 10% glycerol, 50 mM Tris-HCl (pH 7.3), 100 mM NaF, 1 mM EDTA, 1 mM phenylmethylsulfonyl fluoride (PMSF), 1 mM sodium orthovanadate, 1.5 mM magnesium chloride] (Beyotime Institute of Biotechnology, China) was added to the cells at 150 µl buffer per well. Cells were scraped from the wells and disrupted by sonication. After centrifugation at 4°C, extracts (30 µg) of total cellular protein were separated by SDS-PAGE and electrophoretically transferred onto PVDF membranes. The membranes were blocked with 5% non-fat milk in Tris-buffered saline containing 0.1% Tween-20 (TBS-T), followed by incubation with the relevant primary antibody overnight at 4°C, then with the horseradish peroxidase-conjugated secondary antibody for 1 h at room temperature. Immunodetection analyses were accomplished using an eECL Western Blot Kit (CW0049, Cowin Biotech, Beijing, China). Chemiluminescence signals were detected with a lumino-image analyzer (Fluor Chem E, Alpha View, Santa Clara, CA 95051).

### Generation of recombinant adenovirus and infection

The AdEasy System [35] was used to generate recombinant adenovirus expressing NR4A3. In brief, the full-length NR4A3 cDNA containing *Kpn*I and *Xba*I restriction endonuclease sites was subcloned into a pAdTrack-CMV shuttle vector expressing a GFP marker. The positive pAdTrack-NR4A3 recombinant plasmid, linearized with *Pme*I, and an AdEasy-1 adenoviral backbone, were co-transformed into *Escherichia coli* BJ5183 for homologous recombination, and the positive recombined clones were selected according to a previously described protocol. A positive recombined plasmid (pAdEasy-NR4A3) was linearized with *Pac*I, and transfected into HEK293 cells to generate adenovirus that encoded NR4A3. This adenovirus was amplified and purified according to standard techniques. The control adenovirus, Ad-GFP (generated from recombination of the pAdTrack-CMV shuttle vector with AdEasy-1) was amplified and purified in the same way. The expression level of NR4A3 was detected with western blotting after Ad-NR4A3 adenovirus infection of MIN6 cells.

### Generation of expression plasmids for different NR4A3 deletions and production of stably transfected cell lines

Fusion cDNAs containing the full-length NR4A3 coding sequence and a C-terminal-HA epitope tag were cloned into a pLenti6/V5-D-topo vector (Invitrogen). The plasmids of each deletion cDNA (ΔAF1_2–288 aa_, ΔDBD_292–364 aa_, and ΔLBD_398–626 aa_) were generated by mutagenesis techniques. The four recombinant plasmids and a control GFP gene construct were transfected into MIN6 cells, using TurboFect Transfection Reagent (Thermo Scientific). For stable transfection, cells were grown under blasticidin selection (3 µg/ml) for over 15 days, then single clones of cells were picked and amplified. Western blotting analyses were performed to test the stability of the transfected cell lines.

### Statistical analysis

Data are presented as mean ± standard error (S.E.). One-way analysis of variance (ANOVA) and *t*-test were performed using the SPSS 19.0 software package, and p<0.05 was considered statistically significant.

## Results

### TG treatment resulted in NR4A3 expression and UPR activation in MIN6 cells

**Figure 1:**
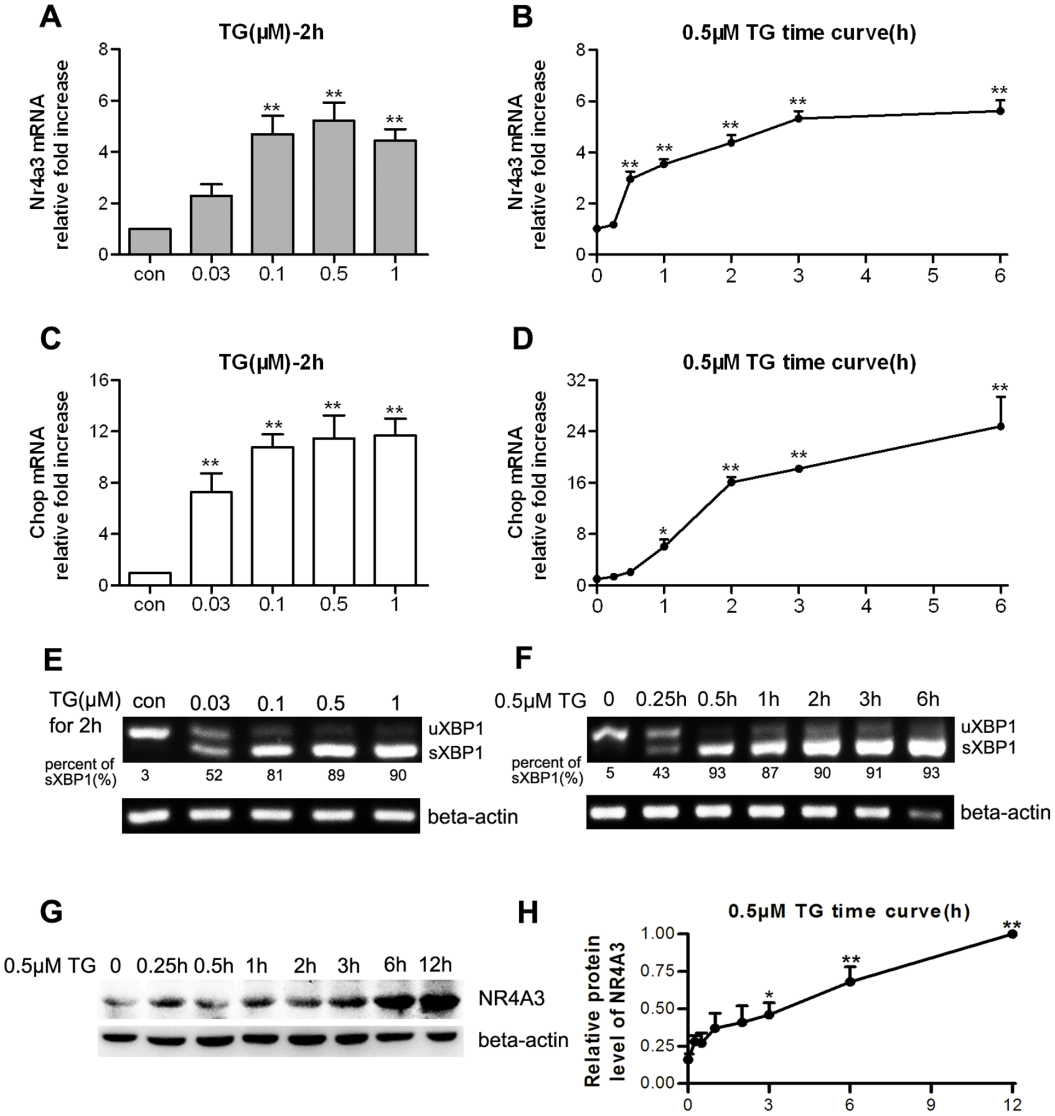
Thapsigargin (TG) treatment induced NR4A3 expression and unfolded protein response (UPR) activation in MIN6 cells. (A, B) NR4A3 mRNA levels in response to (A) different doses of TG and (B) a fixed TG dose at a series of time points. (C, D) *Chop* mRNA levels in response to (C) different doses of TG and (D) a fixed TG dose at a series of time points. Relative mRNA levels of NR4A3 and Chop were determined with real-time quantitative PCR. (E, F) Spliced XBP1 (sXBP1) mRNA formation in response to (E) different doses of TG and (F) a fixed TG dose at different time points. Two forms of XBP1 (a UPR molecule) were detected with reverse transcription PCR. (G) NR4A3 protein profile in response to a fixed TG dose at a series of time points assayed with western blotting. (H) Semi-quantitative analyses of NR4A3 protein in response to a fixed TG dose at a series of time points. Data are shown as mean ± S.E. (n = 4). * p<0.05, ** p<0.01 vs. con (the dissolvent [DMSO] control group) or 0 h (baseline).

MIN6 cells were incubated with different concentrations of TG for 2 h, resulting in NR4A3 mRNA increases, predominantly at doses of 0.1–1 µM (Figure 1A). MIN6 cells treated with a fixed dose of TG (0.5 µM) for a series of time points showed elevation of NR4A3 mRNA and protein levels in a time-dependent manner (Figure 1B, G, H). Meanwhile, Chop mRNA also increased 7–12-fold compared with the control group, from 0.03 to 1 µM (Figure 1C). Time-course experiments showed that in MIN6 cells, Chop mRNA was increased in a time-dependent manner after treatment with 0.5 µM TG (Figure 1D). XBP1 splicing (sXBP1) of 81–90% was detected in MIN6 cells treated with 0.1–1 µM TG (Figure 1E), and sXBP1 mRNA transcription increased markedly in a time-dependent manner after stimulation with 0.5 µM TG (Figure 1F).

### PA treatment resulted in NR4A3 expression and UPR activation in MIN6 cells

**Figure 2:**
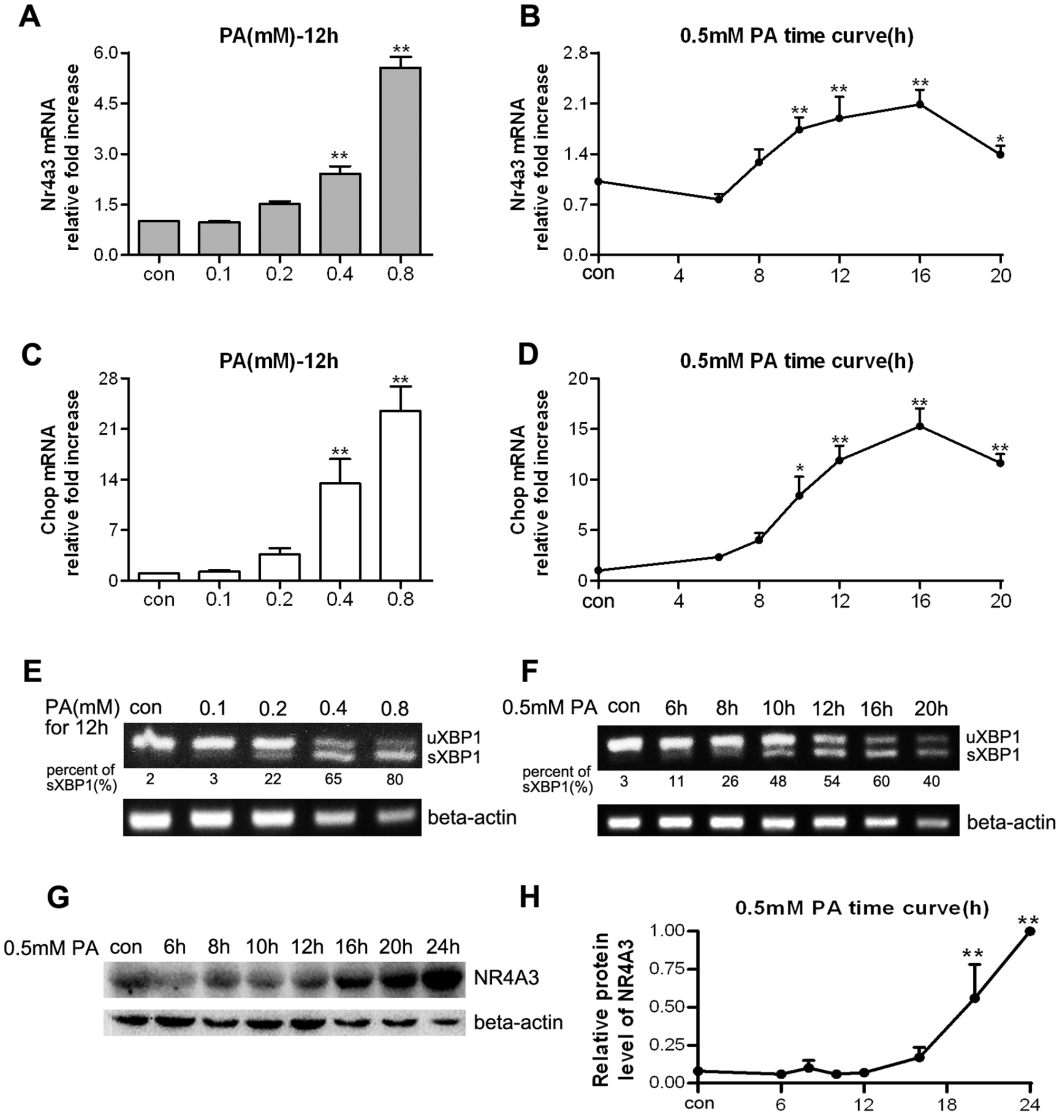
Palmitate (PA) treatment induced NR4A3 expression and unfolded protein response (UPR) activation in MIN6 cells. (A, B) NR4A3 mRNA levels in response to (A) different doses of PA and (B) a fixed PA dose at a series of time points. (C, D) *Chop* mRNA levels in response to (C) different doses of PA and (D) a fixed PA dose at a series of time points. Relative mRNA levels of NR4A3 and Chop were determined with real-time quantitative PCR. (E, F) Spliced XBP1 (sXBP1) mRNA formation in response to (E) different doses of PA and (F) a fixed PA dose at different time points. Two forms of XBP1 (a UPR molecule) were detected with reverse transcription PCR. (G) NR4A3 protein profile in response to a fixed PA dose at a series of time points assayed with western blotting. (H) Semi-quantitative analyses of NR4A3 protein in response to a fixed PA dose at a series of time points. Data are shown as means ± S.E. (n = 4). * p<0.05, ** p<0.01 vs. con. Con is either (A, C, E) the vehicle (0.5% bovine serum albumin [BSA]) or (B, D, F– H) the average basal level in 0.5% BSA at a series of time points.

In MIN6 cells, PA stimulation also enhanced NR4A3 mRNA levels (Figure 2). Treatment with 0.4–0.8 mM PA for 12 h and 0.5 mM PA for 10 to 20 h markedly induced NR4A3 transcription (Figure 2A, B). The trend for Chop mRNA level was similar to that of the induced NR4A3 mRNA in the PA dose and time-course experiments (Figure 2C, D). NR4A3 protein expression was markedly increased upon treatment with 0.5 mM PA in a time-dependent manner in MIN6 cells (Figure 2G, H). Compared with TG treatment, there was a lower level of XBP1 splicing (40–80%) after PA treatment at different doses (Figure 2E). No significant elevation in sXBP1 transcription, and it even decreased in a time-dependent manner after stimulation with 0.5 mM PA (Figure 2F), but XBP1 splicing was clearly observed.

### TM or DTT treatment resulted in UPR activation in MIN6 cells but did not result in enhanced expression of NR4A3

**Figure 3:**
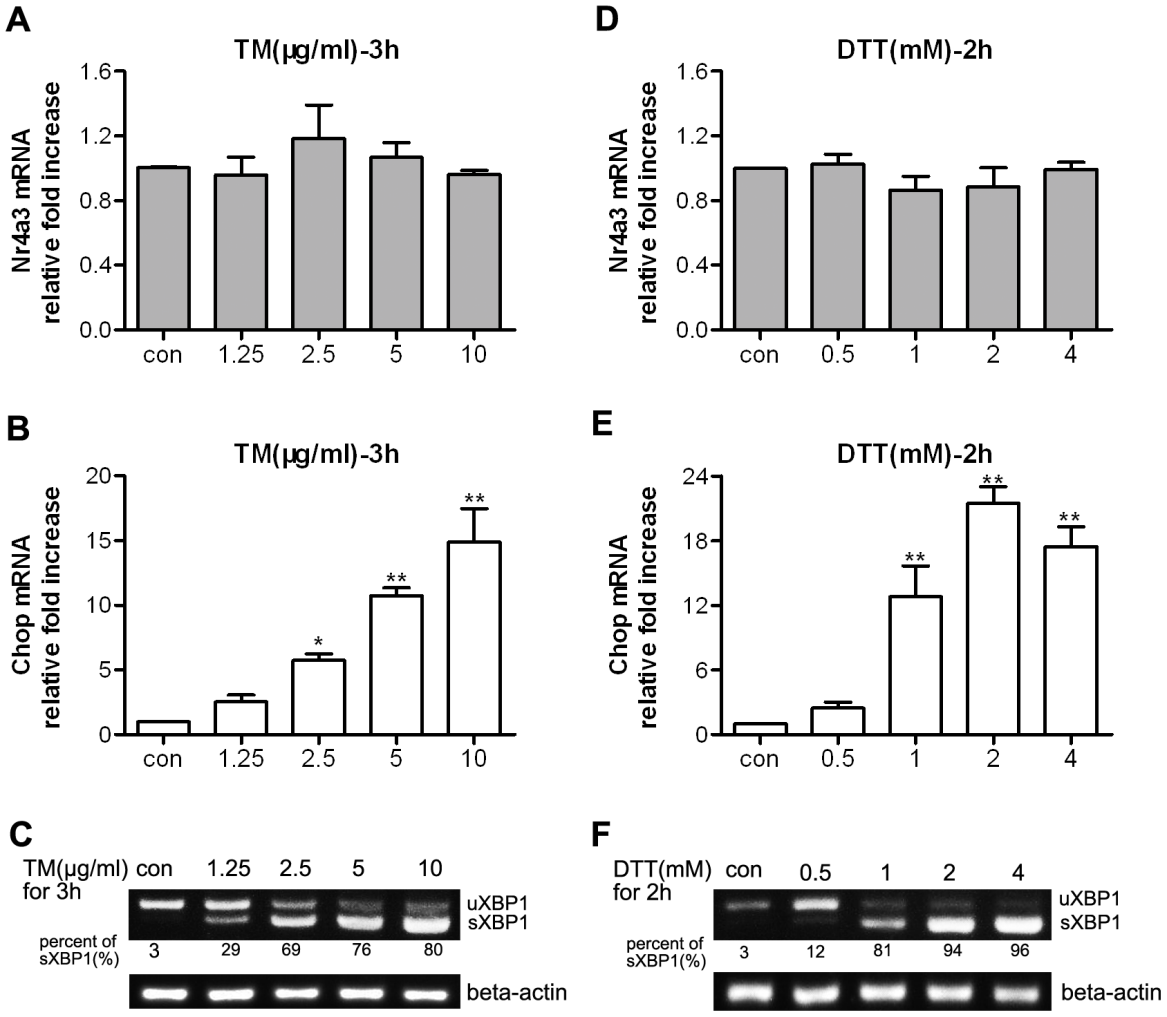
Tunicamycin or dithiothreitol treatment resulted in UPR activation but no NR4A3 increase in MIN6 cells. (A, B) Relative mRNA levels of NR4A3 and *Chop*, respectively, determined with real-time quantitative PCR in response to different doses of tunicamycin (TM) in MIN6 cells. (D, E) Relative mRNA levels of NR4A3 and *Chop*, respectively, determined with real-time quantitative PCR in response to different doses of dithiothreitol (DTT) in MIN6 cells. (C, F) Spliced XBP1 (sXBP1) mRNA formation in response to different (C) TM or (D) DTT doses, respectively. Two forms of XBP1 (a UPR molecule) was detected with reverse transcription PCR. Data are shown as mean ± S.E. (n = 4). * p<0.05, ** p<0.01 vs. con (vehicle control group).

Although TM and DTT treatment triggered obvious ER stress or UPR activation in MIN6 cells, as shown by 69–96% XBP1 splicing and a marked increase in Chop mRNA (Figure 3B, C, E, F), NR4A3 mRNA transcription level was almost unchanged (Figure 3A, D).

### Insulin secretion and insulin gene transcription were negatively correlated with NR4A3 expression levels

**Figure 4:**
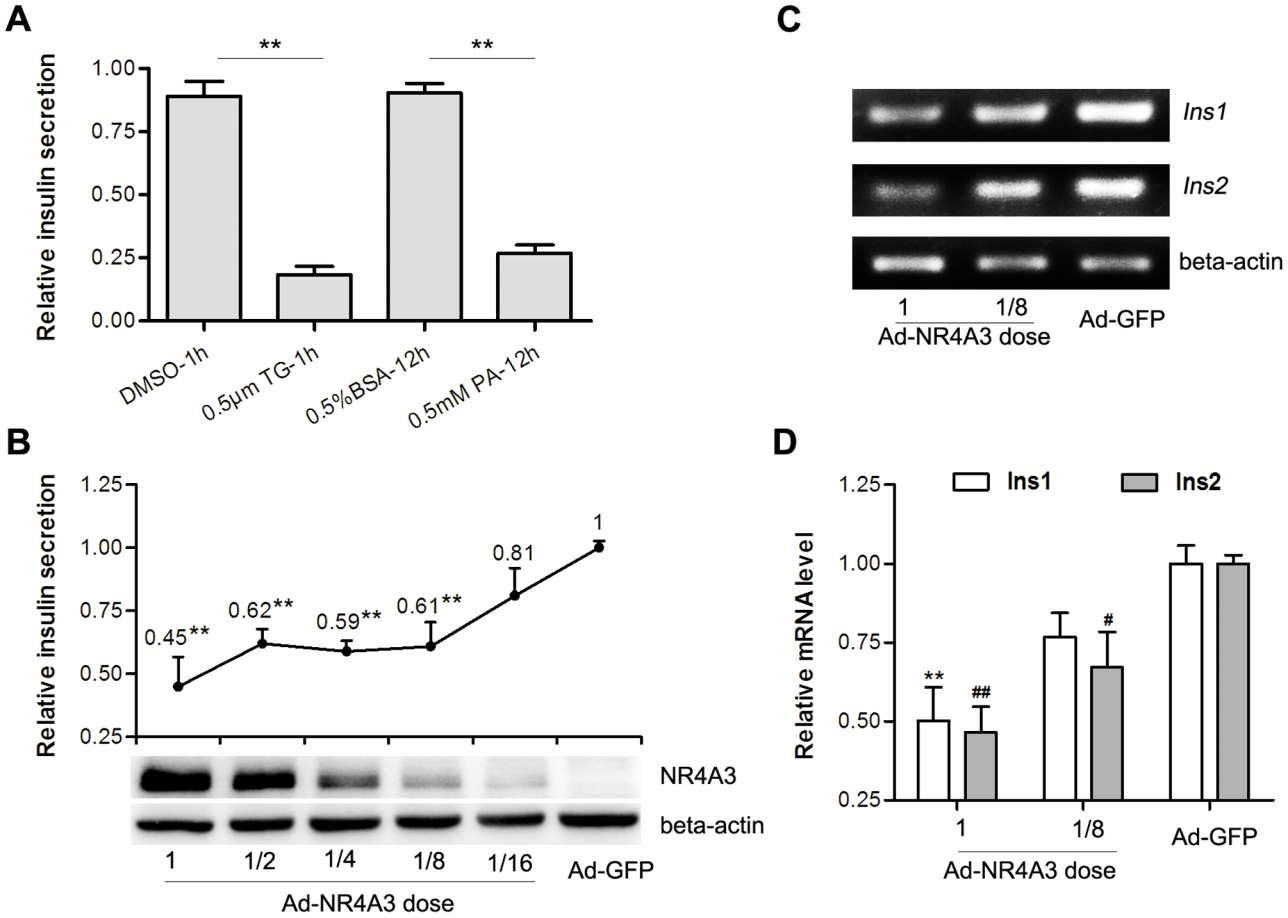
Insulin secretion and mRNA assay in MIN6 cells after infection with equivalent titer of adenovirus. (A, B) Analysis of insulin secretion after glucose stimulation assayed by radioimmunoassay (RIA). (A) MIN6 cells were treated with 0.5 µM thapsigargin (TG) or DMSO (vehicle control) for 1 h, 0.5 mM palmitate (PA) or 0.5% BSA (vehicle control) for 12 h, and the supernatants were assayed for insulin protein level (n = 3). (B) MIN6 cells were infected with a series of double dilutions of Ad-NR4A3 adenovirus (adenovirus encoding NR4A3). Additional Ad-GFP (control adenovirus expressing GFP only) was used for complementary infection in order to ensure each infection had an equal virus titer. Post-infection, levels of secreted insulin were assayed by RIA (n = 4). Western blotting showed NR4A3 protein expression gradually decreasing, and insulin secretion level increasing accordingly. (C) mRNA levels of two insulin genes (*Ins1* and *Ins2*) were determined with reverse transcription PCR in MIN6 cells infected with Ad-NR4A3/Ad-GFP. (D) Semi-quantitative analyses of *Ins1* and *Ins2* mRNA levels in different adenovirus-infected MIN6 cells (normalized to beta actin) (n = 6). Data are shown as mean ± S.E. # p<0.05; **, ## p<0.01 vs. the control group or Ad-GFP infection only.

The effect of the stress inducers TG and PA on insulin secretion in MIN6 cells was assayed by RIA. Treatment of MIN6 cells with 0.5 µM TG for 1 h and 0.5 mM PA for 12 h markedly suppressed insulin secretion (Figure 4A).

The pancreatic beta cell is the unique cell for insulin synthesis. In type 2 diabetes, compensatory excess synthesis of insulin is able to induce stress, especially in the ER-stress [36]. Palmitate-induced NR4A1 was shown to decrease glucose-simulated insulin secretion in pancreatic beta cells [32]. During stress triggered by TG and PA in MIN6 cells, NR4A3 mRNA transcription increased significantly according to the above results (Figure 1, 2). The relationship between NR4A3 expression and insulin synthesis in beta cells was considered worth exploring.

The AdEasy System was used to generate recombinant adenovirus expressing NR4A3. Gradient titer dilutions of Ad-NR4A3 adenovirus were prepared, and MIN6 cells were infected for 44 h (Ad-GFP virus was applied to ensure that every infectation had the same titer of adenovirus), then the level of secreted insulin after stimulation with 25 mM glucose was assayed by RIA (Figure 4B). The relative mRNA expression levels of the insulin genes *Ins1* and *Ins2* were assayed by RT-PCR (Figure 4C, D). The secreted insulin protein and *Ins1*/*Ins2* mRNA levels of the Ad-NR4A3-infected cells were markedly lower than those of the control infected cells (Ad-GFP) (Figure 4B–D). The greater the NR4A3 expression, the lower the level of secreted insulin detected (Figure 4B), thus the secreted insulin and *Ins1*/*Ins2* gene transcription levels were negatively correlated with NR4A3 expression.

### The AF1 and DBD domains of NR4A3 were necessary for down-regulation of insulin gene transcription

**Figure 5:**
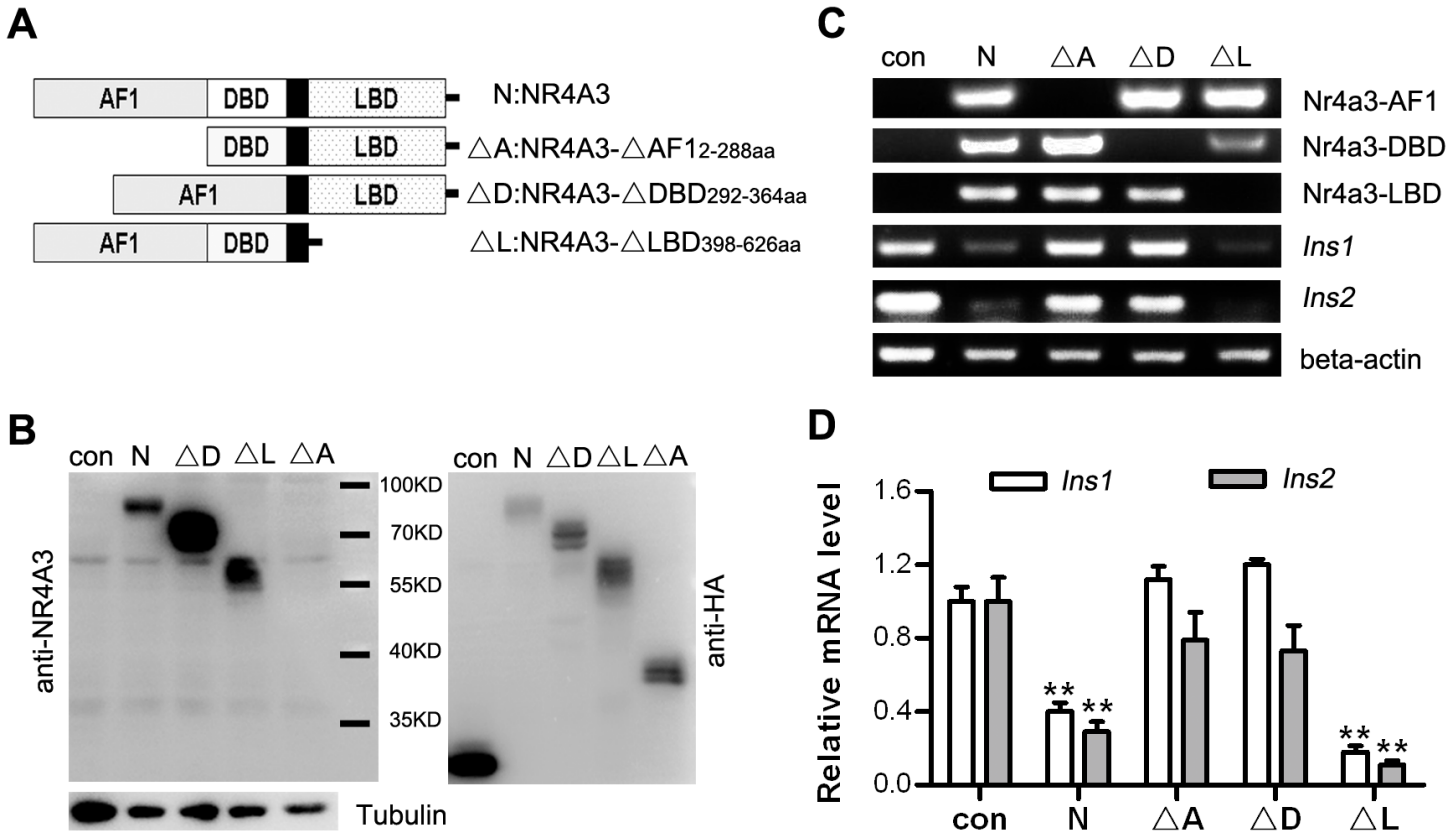
Possible roles of NR4A3 in modulating the expression of insulin genes in MIN6 cell lines. (A) Diagram of constructed wild-type and deletion forms of NR4A3 cDNA. (B) Verification by western blotting MIN6 cell lines stably over-expressing NR4A3 or its deletion forms. (C) mRNA levels of NR4A3 or two insulin genes (*Ins1* and *Ins2*) detected with reverse transcription PCR. Each image is representative of at least three experiments. (D) Semi-quantitative analyses of *Ins1* and *Ins2* mRNA levels normalized to beta actin in various stable cell lines (n = 5). ** *P*<0.01 vs. the control cells. Data are representative of three clone lines. Con, cell line transfected with vector encoding GFP; N, cell line expressing the wild-type NR4A3; ΔA, cell line expressing the 2–288 amino acid (aa) deletion of AF1 (activation function-1 domain); ΔD, cell line expressing the 292–364 aa deletion of the DNA binding domain (DBD); ΔL, cell line expressing the 398–626 aa deletion of the ligand binding domain (LBD). The C terminal of all exogenous genes was HA-tagged to facilitate identification with western blotting.

The NR4A3 cDNA includes the N-terminus activation domain AF1, the DBD, and the C-terminus LBD [2]. According to a previous report, AF1 is the functional domain [37]. In the current study, we constructed three cDNA truncation or deletion vectors (ΔAF1, ΔDBD, and ΔLBD) from full-length NR4A3 (Figure 5A). The cell lines stably transfected with wild-type NR4A3 and the three NR4A3 truncation or deletion mutants were selected with blasticidin antibiotic. The stably control cell line was from the transfectant with parental vector encoding GFP. The C terminal of all exogenous genes was HA-tagged to facilitate identification by western blotting (Figure 5B). *Ins1*/*Ins2* mRNA expression levels were analyzed by RT-PCR, which showed that *Ins1*/*Ins2* expression in NR4A3 wild-type cell lines and ΔLBD cell lines were predominantly lower than in control cells. In the ΔAF1 and ΔDBD cell lines, the mRNA transcription was not significantly different from the control cells (Figure 5C, D). Thus, over-expression of NR4A3 in MIN6 cells down-regulated insulin gene transcription. The modulation of insulin expression by NR4A3 was closely related to AF1 and DBD. Based on these results, NR4A3 acted as a transcription factor to down-regulate the expression of the insulin genes.

**Figure 6:**
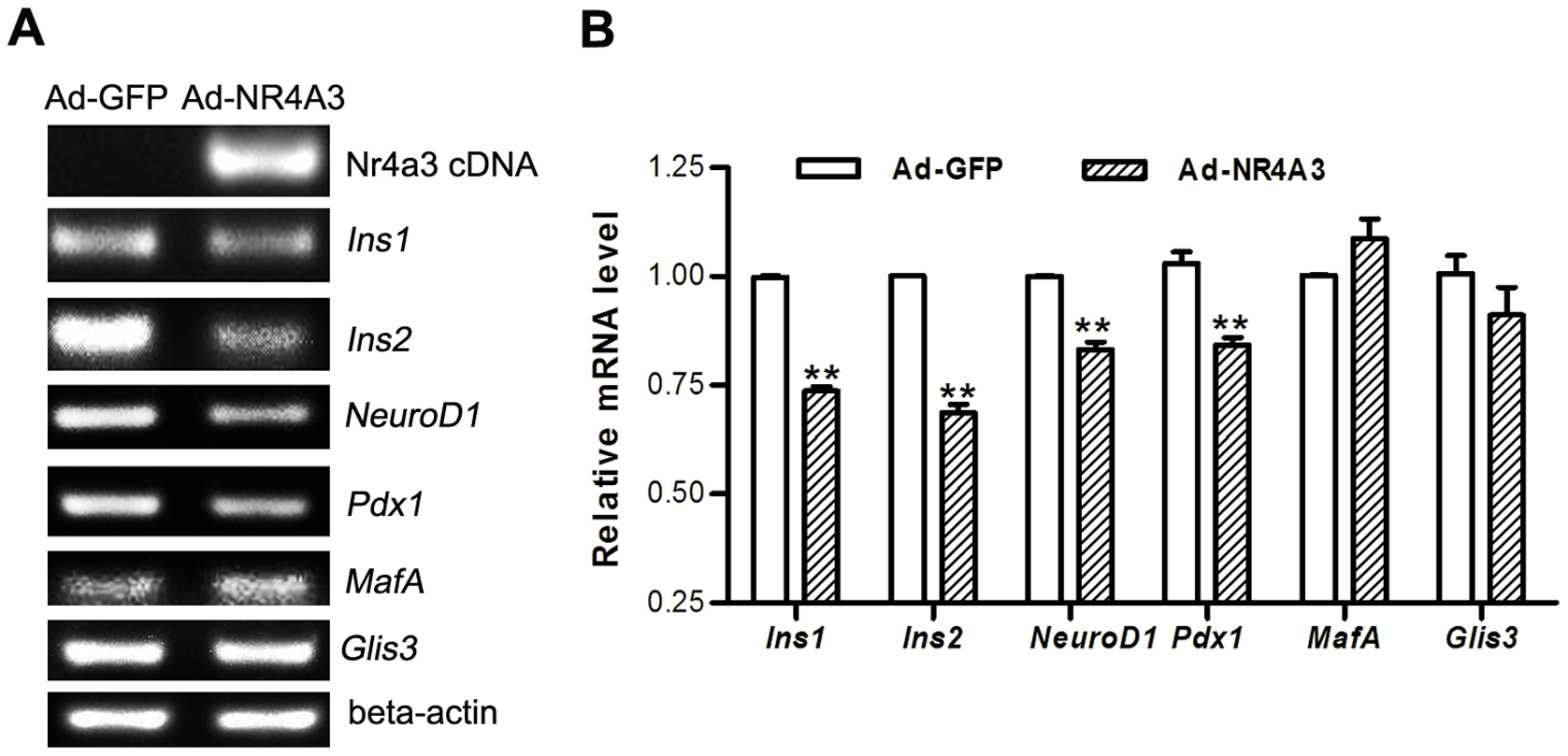
mRNA levels of insulin regulatory genes were analyzed in MIN6 cells infected with adenovirus. (A) mRNA levels of two insulin genes or insulin transcription regulatory genes assayed by reverse transcription PCR. Data were representative of at least three experiments. (B) Quantitative analyses of mRNA levels by real-time quantitative PCR. Data are shown as mean ± S.E. (n = 4). ** p<0.01 vs. Ad-GFP infection.

Inhibition of insulin transcription by NR4A1 in MIN6 cells was indirect [32]. In order to explain the manner of NR4A3-regulated insulin expression, we analyzed the changes in transcription factor binding to the insulin gene promoter in MIN6 cells infected with Ad-NR4A3. *Pdx1* and *NeuroD1* mRNA levels in Ad-NR4A3-infected cells were significantly lower than in control infected cells (Figure 6A, B). Both *Pdx1* and *NeuroD1* are positive transcription factors, which bind to the insulin gene promoter [38], [39]. Unlike with *Pdx1* and *NeuroD1*, the mRNA levels of *MafA* and *Glis3* (the other two positive transcription factors for insulin [40]–[42]) showed no significant changes after adenovirus-mediated over-expression of NR4A3 protein (*P*>0.05).

## Discussion

NR4A3, one of orphan nuclear receptors, has no currently identified natural or synthetic ligands, because of its special LBP structure [5]. It was shown that the AF1 domain of NR4A3/NOR-1 mediated the trans-activation, coactivator recruitment, and activation functions [37]. Many stimuli can induce NR4A mRNA transcription in different cell types, such as cancer cells (BGC-823 and HepG2) [20], [25], neuronal cells [7]–[10], fibroblasts (NIH3T3 and L1) [14], pancreatic beta cells (INS1, MIN6) [16], [32], vascular smooth muscle cells [17], and T cells [34]. In the current study, both TG and PA resulted in elevated NR4A3 mRNA transcription and protein expression. TG is a highly potent and specific inhibitor of the Ca^2+^-dependent ATPase in the ER, which leads to an efflux of Ca^2+^ from the ER lumen into the cytosol and a sustained depletion of the ER Ca^2+^ pool [15], resulting in classic ER stress and UPR activation. XBP1 mRNA splicing to a 26 bp shortened form (sXBP1) is one of the representative molecules produced as a result of UPR [43]. Chop, a proapoptotic transcriptional regulator, also appears in ER stress and stress-inducing apoptosis [25]. Our data showed that TG treatment resulted in >90% XBP1 splicing and markedly increased Chop mRNA expression, while simultaneously, NR4A3 transcription and translation also significantly increased in MIN6 cells (Figure 1). Long-chain free fatty acid (FFA) could induce NR4A1 transcription immediately in INS1 beta cells [16]. In the current study, PA long-time incubation (10–24 h) induced an increase in NR4A3 mRNA and protein in MIN6 cells (Figure 2A, B, G, H). The mechanism of PA treatment was different from that of TG stimulation in MIN6 cells, because of less XBP1 splicing form (40–80%) detected, and no significant time-dependent increase in transcription of sXBP1 (Figure 2E, F). However, both TG and PA treatment markedly elevated Chop mRNA levels. Two other inducers of ER stress, TM (an inhibitor of protein glycosylation) and DTT (which disrupts or prevents protein disulfide bonding) [44], were able to induce ER stress and UPR activation (as shown by XBP1 splicing and Chop mRNA elevation), but were not able to change NR4A3 mRNA level (Figure 3). We do not know the exact reason why TG and PA are able to induce NR4A3 expression while TM and DTT are not, but we know that both TG and PA induce ER stress by specific mechanisms related to sustained ER Ca^2+^ depletion. It was reported that the NR4A1/Nur77 promoter could be regulated by Ca^2+^-related molecules [15], therefore, our presumption is that the elevated NR4A3 transcription might be related to TG or PA-induced ER Ca^2+^ efflux. We conclude that in MIN6 cells, elevation of Ca^2+^ in the cytoplasm, rather than ER stress, is the critical factor in NR4A3 induction. NR4A3 elevation and UPR activation are two separate cell responses to some adverse conditions.

We found that when MIN6 cells were treated with 0.5 µM TG or 0.5 mM PA, insulin secretion was markedly reduced (Figure 4A). It has been reported that as a transcription factor, NR4A1/Nur77 inhibited insulin secretion and transcription in MIN6 cells [32]. The relationship between NR4A3 over-expression and the expression or secretion of insulin in pancreatic beta cells remains unknown. We examined the effect of NR4A3 on insulin gene transcription and secretion in MIN6 cells, and found that the mRNA levels of two insulin genes (*Ins1* and *Ins2*) were reduced after adenoviral infection with Ad-NR4A3, and this reduction was dependent on the NR4A3 adenovirus titer (Figure 4C, D). We also found that NR4A3 protein levels were negatively correlated with glucose-induced insulin secretion in MIN6 cells infected with different titers of adenovirus encoding NR4A3 (Figure 4B).

We further examined whether NR4A3 was able to reduce insulin transcription or secretion by changing the trans-activation of some genes. We found that deleting the activation domain AF1 or the DBD of NR4A3 did not affect insulin gene transcription, indicating that the effect of NR4A3 in reducing insulin production was related to its transcription activity (Figure 5C, D). We tested the changes of four positive transcription factors of insulin gene, and found that the mRNA levels of *NeuroD1* and *Pdx1* were significantly decreased and that of *MafA* and *Glis3* were unchanged (no statistical significance) after over-expression of NR4A3 by adenoviral infection (Figure 6A, B). These data indicated that NR4A3 modulates insulin gene transcription indirectly. There are two contradictory conclusions about NR4A subfamily regulating insulin in pancreatic beta cells currently. Ordelheide AM found NR4A3/Nor-1 may directly bind to the insulin gene and up-regulate its transcription and secretion in rat pancreatic beat cells INS-1 [45]. But in Briand O's research, adenoviral over-expression of NR4A1/Nur77 was shown to inhibit insulin transcription and secretion in mouse pancreatic beta cells MIN6 indirectly [32]. Our results showed that NR4A3 was identified as a negative regulator of insulin gene expression in mouse beta cells MIN6. We also found adenoviral over-expression of NR4A1/Nur77 in MIN6 cells resulted in decreased expression and secretion of insulin (data not shown). Nur77 null mice displayed markedly higher fasting insulin levels, which revealed the relationship between NR4A1 and insulin expression *in vivo* [46]. The contradictory results of NR4A subfamily regulating insulin expression and secretion may be due to different cell lines with different background. The mechanism need to be further studied.

**Figure 7:**
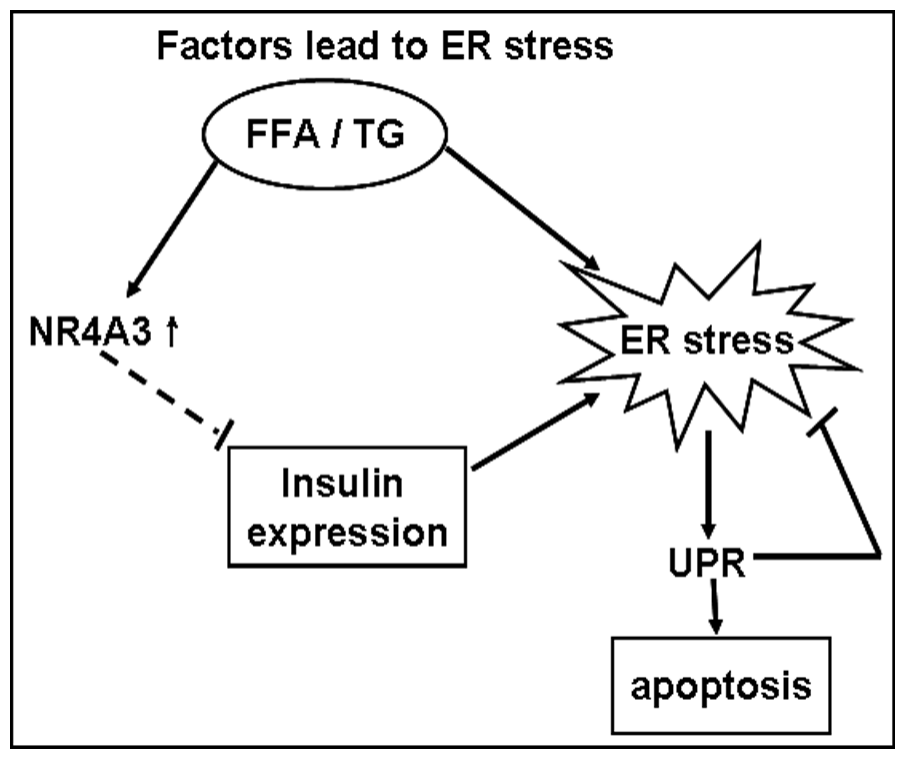
A model for possible role of NR4A3 in releasing pancreatic beta cells ER stress. Upon expression and activation of NR4A3 induced by factors such as long-chain free fatty acids (FFA) and thapsigargin (TG), which lead to ER stress, unfolded protein response (UPR) activation, and even apoptosis, this orphan nuclear receptor decreases insulin expression, which indirectly releases the burden of ER.

According to our results, we therefore hypothesize that elevated expression of NR4A3 or its similar family members can down-regulate insulin expression in mouse pancreatic beta cells MIN6, which in turn reduces the burden of ER in the pancreatic beta cell (Figure 7).

## Supporting Information

Table S1 Sequence information of the primers used for RT-PCR/QPCR. F: forward primer. R: reverse primer.
(DOC)
