# Supplementary material for: Elevation of NR4A3 Expression and Its Possible Role in Modulating Insulin Expression in the Pancreatic Beta Cell
Source: PLoS One. 2014 Mar 17;9(3):e91462. doi: 10.1371/journal.pone.0091462 (PMC3956668; doi:10.1371/journal.pone.0091462)
Supplement: Table S1 — Sequence information of the primers used for RT-PCR/QPCR. F: forward primer. R: reverse primer. (DOC) [file pone.0091462.s001.doc]

**Table S1. Sequence information of the primers used for RT-PCR/QPCR.**

| gene | sequence | Product size (bp) |
| --- | --- | --- |
| Mouse β-actin | F: gtgacgttgacatccgtaaaga | 245 |
|  | R: gccggactcatcgtactcc |  |
| Mouse NR4A3 | F: aggattcactgatctccccaa | 140 |
|  | R: gatgcaggacaagtccattgc |  |
| Mouse Chop | F: ctggaagcctggtatgaggat | 121 |
|  | R: cagggtcaagagtagtgaaggt |  |
| Mouse XBP1 | F: aaacagagtagcagcgcagactgc | 480(uXBP1) |
|  | R: tccttctgggtagacctctgggag | 454(sXBP1) |
| Mouse Ins1 | F: aacagcaaagtccagggggca | 275 |
|  | R: agaagaagccacgctccccaca |  |
| Mouse Ins2 | F: cttcttctacacacccatgtccc | 101 |
|  | R: ccaaggtctgaaggtcacctg |  |
| Mouse Pdx1 | F: cctttcccgaatggaaccga | 228 |
|  | R: gggccgggagatgtatttgt |  |
| Mouse NeuroD1 | F: cggactttcttgcctgagca | 155 |
|  | R: ggcttgacgtggaagacgtg |  |
| Mouse MafA | F: aggaggaggtcatccgactg | 113 |
|  | R: cttctcgctctccagaatgtg |  |
| NR4A3-AF1 | F: cccggtaccatgccctgcgtccaagcccaatatag | 243 |
|  | R: ccgctgcatttggtacacg |  |
| NR4A3-DBD | F: ccactagtcatggagggcacgtgtgccgtgtg | 273 |
|  | R: ctttggtttggaaggcagacgacc |  |
| NR4A3-LBD | F: gatagtctgaaagggaggagaggtcg | 186 |
|  | R: agcagcctggtcagtggg |  |

F: forward primer. R: reverse primer.
